# Coaxial Mach–Zehnder Digital Strain Sensor Made from a Tapered Depressed Cladding Fiber

**DOI:** 10.3390/s22197145

**Published:** 2022-09-21

**Authors:** Sergio Celaschi, Nicolas Grégoire, Younès Messaddeq, Claudecir R. Biazoli, Gilliard N. Malheiros-Silveira

**Affiliations:** 1 Centro de Tecnologia da Informação Renato Archer, Campinas 13069-901, Brazil; 2 Centre d’Optique, Photonique et Laser, Université Laval, Quebec City, QC G1V 0A6, Canada; 3 Department of Communications, School of Electrical and Computer Engineering, University of Campinas (UNICAMP), Campinas 13083-852, Brazil

**Keywords:** digital optical sensors, strain sensor, fiber optics components, Mach–Zehnder interferometer, fiber optics

## Abstract

An in-line digital optical sensor was proposed. It was built from a tapered depressed-cladding single-mode fiber and modeled as a coaxial Mach–Zehnder interferometer. The principle of operation of the optical digital sensor is based on the computation of the number of optical power transfer turning points (PTTP) from the transmission data of the component. Biconic tapers with high values of PTTP, high spectral resolution, high extinction ratio, and low insertion loss were modeled, fabricated, and characterized. As a proof of concept, an in-line digital strain sensor was fabricated and characterized. It presents a free spectral range of 1.3 nm, and produced 96 PTTP, at *λ*_0_ = 1.55 μm, under stretch of Δ*L* = 707 µm, therefore producing a digital resolution of 7.4 µm/PTTP. The sensor also produced a quasi-symmetric response to stretch and compression.

## 1. Introduction

Optical devices based on fiber tapers (FT) encounter diversified practical uses in a broad range of applications [1,2]. In optical communication, they can be used as passive notch filters [3,4], acousto-optic tunable filters [5], super-continuum light generation [6], tunable fiber lasers [7], to name a few. In sensing, they can be used to probe a myriad of measurands: physical (strain [8,9,10], stress [11,12], force [13], pressure [14], angle [15,16] and temperature [17,18,19]), chemical [20,21], bio [22], etc. Those FTs may be fabricated by different processes, most frequent, stretching a heated length of commercial single-mode fiber is the preferable one because of the cost and simplicity of fabrication.

Fiber Optic Analog Sensors (FOAS) are crucial to several applications as mentioned above. The intrinsic bandwidth of such sensors offers distinct advantages in their ability to probe and transport the resultant signal, in addition to being lightweight and small size. The FOAS may be modeled by assuming different mechanisms of operation, and modal interferometry, may produce the most sensitive devices, but they have a drawback: they present a very low linear dynamic range due to their intrinsic sine square transfer function. As well-known, tapering a communication single mode fiber may allow a spectral transmission exhibiting a sine square spectral oscillatory behavior, which can be characterized by a free spectral range (FSR). As previously reported in the literature [4], the tapering of a single-mode depressed-cladding fiber (DCF), or W-type fiber, with adiabatic fiber prolife [4,23,24], may result in only two super-modes HE_11_ and HE_12_ propagating along it. In contrast to using a commercial fiber such as SMF-28 under the same tapering conditions, the DCF may produce low-loss transmission, large modulation depth, and very short FSR [4]. In general lines, the HE_11_ and HE_12_ creation-annihilation at the phase matching regions and their transmission at the taper waist length behave like a coaxial Mach–Zehnder interferometer (CMZI) [4,9,12,17]. The principle of operation of this optical component as a sensor can occur in two ways widely explored in literature: by sensing its optical length, or probing its external refractive index [2]. However, when properly designed, the FT can be seen as an all-optical intrinsic digital sensor enabled to probe external force fields. To the best of our knowledge, such an approach has not been reported in the literature up to now. 

In this manuscript, we propose an all-optical all-fiber digital sensor based on a biconic taper. In comparison to FOAS, it can also be applied to probe the external refractive index or the variation of the optical length/path, but, in contrast, the way the output signal is observed is intrinsically digital. As a proof of concept, we demonstrate the sensor operation as a digital strain sensor using an in-line taper based on DCF. In this case, the principle of operation is based on sensing the optical length of the component. As an example of preliminary experimental result, 96 optical power transfer turning points (PTTP) were produced, at *λ*_0_ = 1.55 μm, when the sensor 104 mm long, with FSR = 1.3 nm, was stretched by Δ*L* = 707 µm, thus producing a digital resolution of 7.4 µm/PTTP. The results also demonstrated a quasi-symmetric response to stretch and compression. For comparison’s sake, the same sensor produced an analog spectral resolution (1/2 FSR = 1 PTTP) of about 0.7 nm, thus demonstrating the advantage of using this special DCF as either digital (our proposal) or analog (standard approach) in-line all-fiber sensor. Additionally, those results do not mean the limit of resolution of this sensor was reached, since the sensibility is increased as the FSR gets smaller [25]. This type of taper, assuming diameters ranging from 0.32 to 0.51 µm, was reported in [26]; those waist dimensions can reach FSR values in the pm scale, i.e., values one order of magnitude lower than the mentioned in our preliminary results reported here, revealing that even higher resolutions are possible. 

## 2. Materials and Methods

### 2.1. Characteristics of the Highly DCF

**Figure 1:**
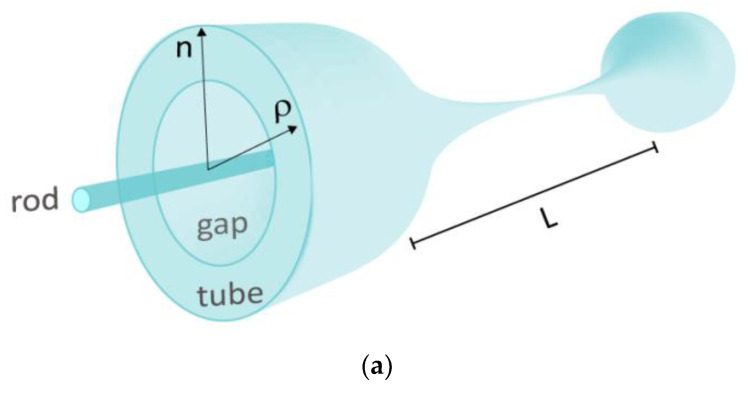

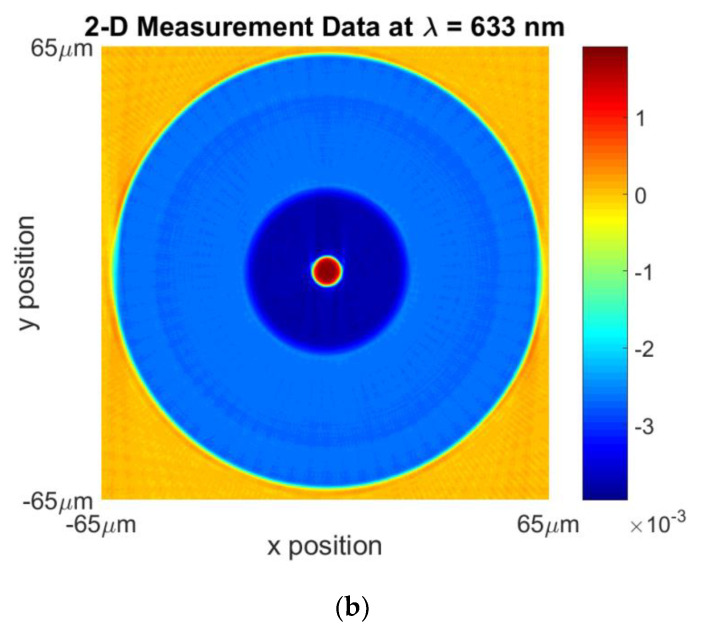
(**a**) 3-D schematics of a biconic FT based on a DCF. *L* represents the biconic taper elongation length, *ρ* and *n* are the radii and the refractive indexes of each DCF layer (rod, gap, tube), respectively. The refractive index in each layer is highlighted at the taper end’s face. (**b**) 2-D experimental data of the refractive index distribution from the DCF used in this work.

The initial geometric (layer radius, *ρ*) and optical (refractive indexes, *n*) parameters for each layer (rod, gap, and tube) of the highly DCF are presented, as a schematic, in Figure 1a, where one of the taper end’s faces highlight its refractive indexes distribution. The values of these parameters used in this study, before tapering the fiber are: *ρ_rod_* = 4.5 µm, *ρ_gap_* = 22 µm, *ρ_tube_* = 62.5 µm, *n_rod_* = 1.4485, *n_gap_* = 1.4428, *n_tube_* = 1.4440, *n_external_* = 1. Figure 1b shows the refractive index distribution of this fiber measured at *λ*_0_ = 633 nm, by the equipment IFA-100 Fiber Index Profiler Version 10.0, located at COPL/ULAVAL, where the indexes contrast is evident. In the non-tapered fiber, its core (or rod) is single mode starting at *λ*_0_ = 1.45 µm to longer wavelengths. 

**Figure 2:**
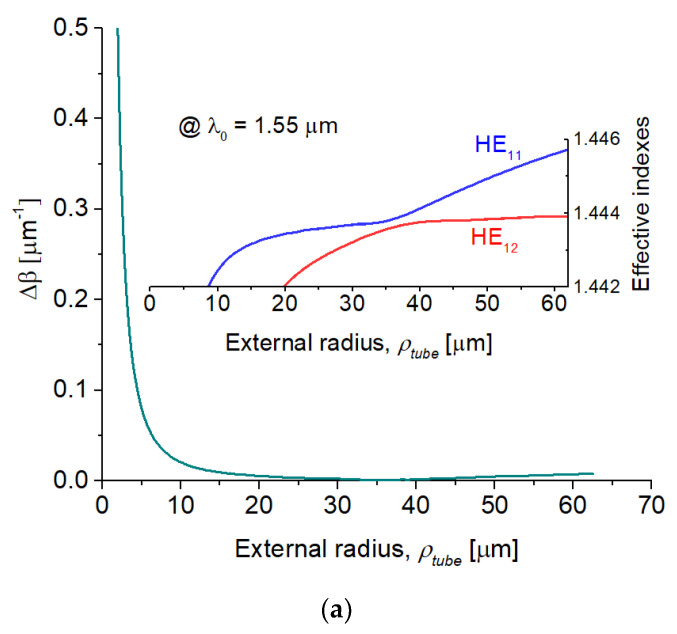

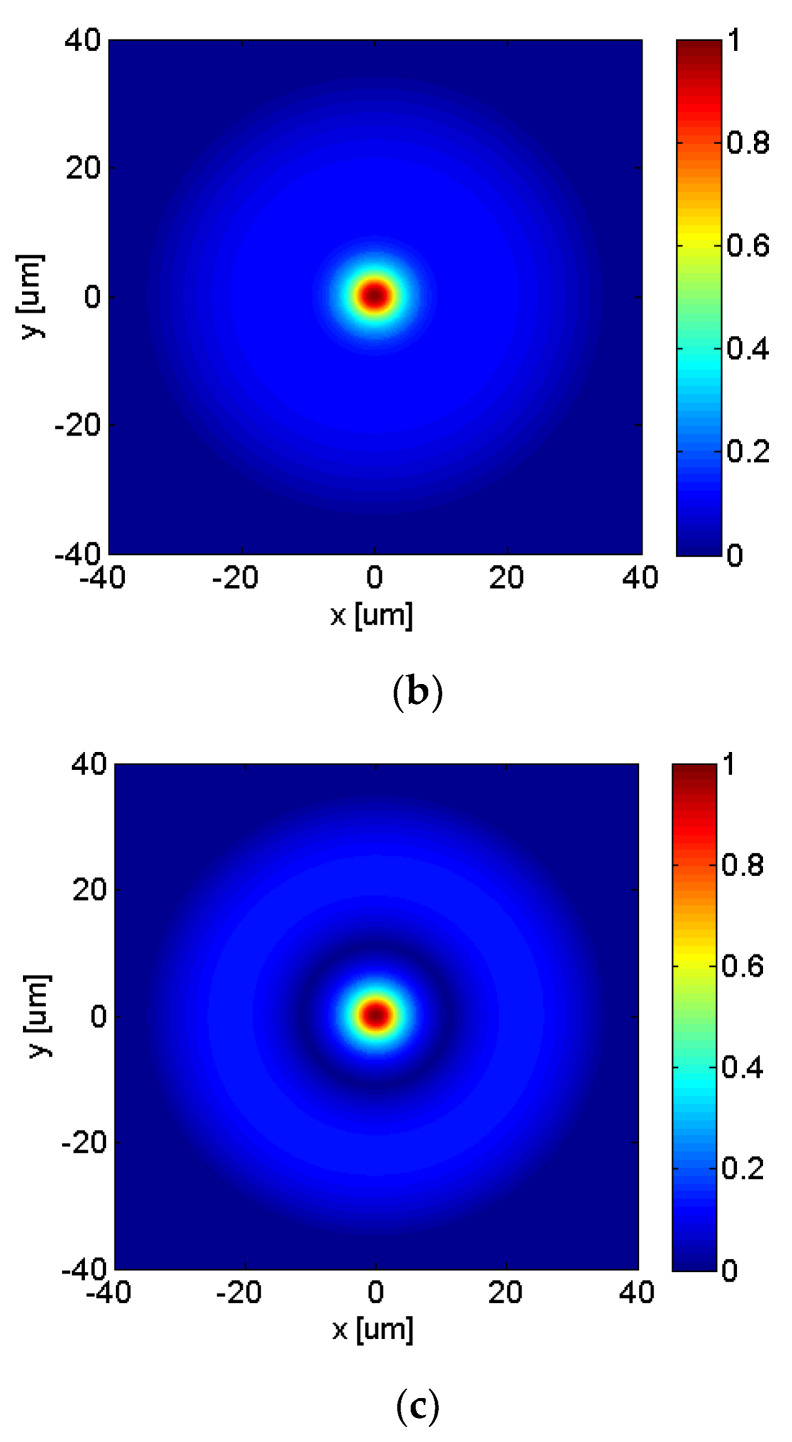
(**a**) ∆*β* of the modes HE_11_ and HE_12_ during *ρ_tube_* variation at *λ*_0_ = 1.55 μm. Inset outlines the effective refractive indexes near the phase-matching. (**b**,**c**) show the normalized modulus of the E-field for the modes HE_11_ and HE_12_, respectively, at the phase matching condition.

The numerical calculation of the optical properties from the propagating modes involved during the process was performed by means of the frequency-domain finite element method [27]. For this simulation, the ratio of the DCF layers along the taper regions is supposed to remain constant during the tapering. Figure 2a shows the dependence of the difference between the propagation constants, ∆*β* = *β*_1_ − *β*_2_, versus the external radius variation. *β*_1_ and *β*_2_ are, in this case, the respective propagation constants of the modes HE_11_ and HE_12_. The phase matching between the modes HE_11_ and HE_12_ occurs at *ρ_tube_* ~ 36 µm as shown in the inset. Additionally, according to our simulations, the HE_12_ mode cut-off occurs at *ρ_tube_* ~ 1 µm. Figure 2b,c shows the normalized modulus of the E-field for the modes HE_11_ and HE_12_, respectively.

### 2.2. Modeling of the Biconic Taper Profiles

The modeling of such taper profiles, as depicted in Figure 1a, can be obtained from two coupled partial differential equations. The mass conservation is governed by the continuity Equation (1), and the axial-momentum conservation by the 1-D Equation (2) [4,28]:

(1) $$\frac{\partial }{\partial t}A_{n}(z,t)+\frac{\partial }{\partial z}\left(A_{n}(z,t)v(z,t)\right)=0,$$

(2) $$\frac{\partial }{\partial z}\left(\eta (z)A_{n}(z,t)\frac{\partial }{\partial z}v(z,t)\right)=0.$$

These equations relate the axial velocity, *v(z*,*t)*, and the normalized cross-section area of the taper, *A_n_(z*,*t) = ρ*^2^*_tube_(z,t)/r_o_*^2^. The temperature profile of the heating element, *T(z)*, the total time, *t_T_*, the pulling velocity, *v_o_*, and the taper elongation length *L*, determine the taper profiles. We assumed the axial viscosity, *η(z)*, of the fiber material to be uniform, and composed of pure SiO_2_. 

**Figure 3:**
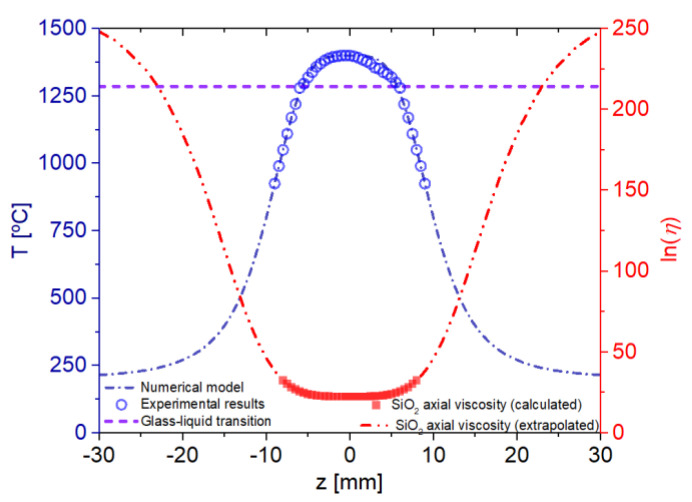
Experimental and modeled temperature curves of the graphite tube (heating element). The SiO_2_ glass liquid transition temperature is shown in (dash line). Axial viscosity of the fiber material is shown in squares (red color). Below 1000 °C, extrapolation follows the temperature curve model of the graphite element.

Figure 3 shows the graphite heater temperature curve (a cylindrical tube 28 mm long). The temperature profile (in Celsius) numerical and experimental are represented respectively in dark-blue dash-dot line and open circle. The axial viscosity *η(z)* is presented in the red dash-dot line and solid squares.

The temperature profile model from the heating element is obtained by fitting the experimental profile with the following expressions:

(3) $$T\left(z,z_{o}\right)=T_{o}+\frac{T_{h}-T_{o}}{1+{\left(\frac{\left|z\right|}{z_{o}}\right)}^{2b}},$$

(4) $$z=z_{o}{\left[\frac{T_{h}-T_{g}}{T_{h}-T_{o}}\right]}^{\frac{1}{2b}}.$$

where *T_o_* = 200 °C, *z_o_* = 10, *T_h_* = 1400 °C, *T_g_* (glass-liquid transition temperature) = 1285 °C, and *b* = 2. The axial viscosity curve of the SiO_2_ fiber material shown in Figure 3 was obtained by fitting data from [29], down to 1000 °C, using the following equation:

(5) $$\left(z,z_{o}\right)=e^{\;\frac{c}{T{\left(z,zo\right)}^{1.28}}}$$

where *c* = 2.4 × 10^5^.

### 2.3. Adiabatic Taper Profile

In slowly varying waveguides, the total E-field can present variations along a distance equals to the beat length, *z_b_* = 2Δ/(*β*_1_ − *β*_2_). Thus, in order to assure the local mode solution, the non-uniformities of the guide should occur over a distance larger than *z_b_*. Thus, this adiabatic condition is respected when the waveguide radius does not vary, significantly, along a distance *z_b_* [23]:

(6) $$\frac{1}{\rho_{tube}\left(z,t_{final}\right)}\frac{\partial \;\rho_{tube}\left(z,t_{final}\right)}{\partial z}<\frac{1}{z_{b}}\;,\;\;\;\;$$

**Figure 4:**
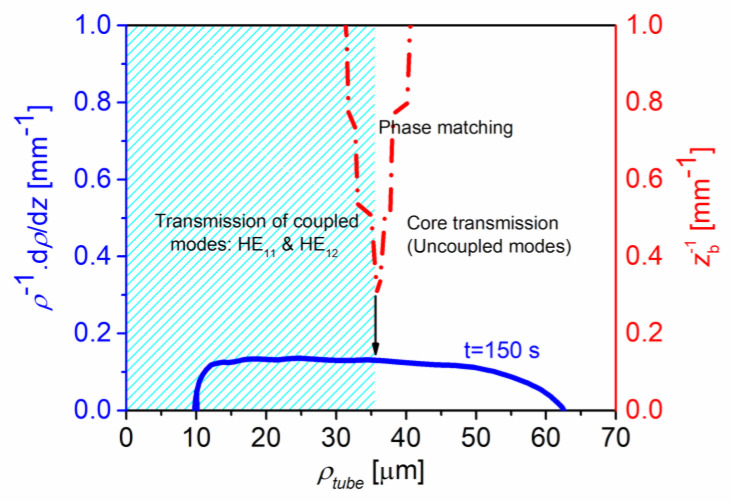
Normalized slope of the taper (blue solid line) and 1/*z_b_* (dashed point red line) versus outer radii, *ρ_tube_*, for the elongation time, *t_T_* = 150 s.

The shape of the tapered region is determined by the dynamics of the fiber heating-and-pulling process, and the temperature profile of the heating element. The FT profiles were measured using an optical microscope, also modeled numerically [4]. Figure 4 shows that the first term of the relation (6) is below the second one. Therefore, confirming the taper profile fabricated obeys the adiabatic slowness criterion [24].

### 2.4. All-Fiber CMZI

**Figure 5:**
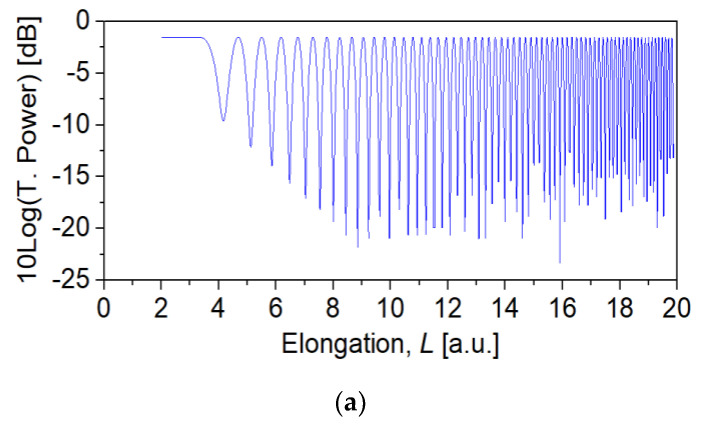

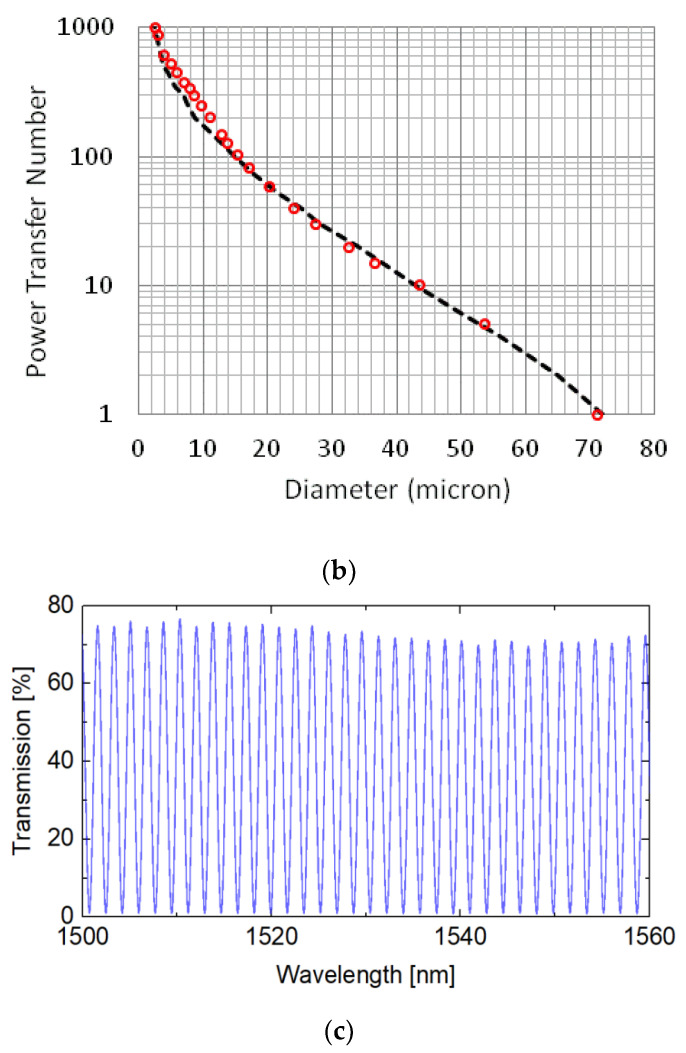
(**a**) Transmitted power recorded at *λ*_0_ = 1.55 μm during taper fabrication for the first 65 oscillations. (**b**) PTN versus taper waist diameter. The experimental results are represented in red open circles, and the model simulation by black dashed line. According to the simulation, longer tapers (*L* ~ 85 mm) result in PTN ~ 10^3^, 1.4 < FSR < 1.8 nm, and 4 < *ρ_waist_* < 6 µm. (**c**) Spectral response obtained from a taper 76 mm long, FSR = 1.7 nm, taper waist diameter ~5.6 µm, and insertion loss ~1.1 dB.

Figure 5a shows the first 65 power oscillations recorded at *λ_0_* = 1.55 μm from the fiber core output during the taper fabrication. The number of times the transmitted power drops, and returns to its maximum value is named power transfer number (PTN). Additionally, the PTN of such a device assume higher values as the taper elongation increases. Figure 5b shows the PTN as a function of the taper external diameter, producing up to roughly 10^3^ PTNs.

The PTN affects dramatically the spectral response of such tapers, i.e., tapers with higher PTNs produce higher resolution FSR. For example, a taper 76 mm long showing PTN = 907, at *λ*_0_ = 1.55 μm, produced FSR ~1.7 nm. The measured spectral response of this taper is shown in Figure 5c, which presents an insertion loss ~1.1 dB.

## 3. Results and Discussion

As shown in the previous session, the specific refractive index profile and adiabatic tapering condition of this DCF could produce high PTNs and high-resolution spectral filters; not easily reproduced or obtained using standard commercial fibers. We use these characteristics to demonstrate a digital linear strain sensor in this session.

The output power of this FT experiences, at a fixed wavelength, a number of PTTP when it is under stress, and this PTTP is directly proportional to Δ*L/L_c_*. An intrinsic sine square transfer function has two PTTP per cycle. We modeled the axial strain, *ε =* Δ*L/L_c_,* where *L_c_* is the intra clamps length, on the optical length of the interferometer, by applying a strain *σ = F/π·r^2^(z,t_T_)* [N/m^2^] at the FT clamp points. *F* is the applied external force. For approximation’s sake we derived Equation (7) from Hooke’s Law, and assumed a Young’s Modulus of *r =* 7 × 10^10^ N/m^2^ for SiO_2_.

(7) $$∆L=\left(\frac{\sigma_{e}\;}{\gamma }\right)*\int_{-L_{c}/2}^{+L_{c}/2}\frac{dz}{r{\left(z,t_{T}\right)}^{2}}\;\;\;$$

To preserve the sensor integrity, the elastic limit of the SiO_2_, of the FTs were restricted to an axial stress of *σ* ~ 5 × 10^6^ N/m^2^, i.e., 0.007% of the Young’s Modulus of the SiO_2_. From the solution of Equations (1) and (2), an unstressed FT ~ 80 mm long has a minimum radius at the waist 3 < *ρ_waist_* < 4 μm, and PTN ~840 at *λ*_0_ = 1.55 μm. According to Equation (7), when this FT is submitted to *σ_e_*, it will be elongated by Δ*L* ~700 µm, Δ*L/L_c_* ~ 0.7%, which is equivalent to an increase ΔPTN ~ 50, or a number of PTTP ~ 100. 

### Case Study: In-Line All-Fiber Digital Optical Strain Sensor

For this case study, we fabricated a biconic taper using the same DCF, but by using a different fused and pull process, known as the flame brushing technique [30], in special, to explore the advantage of this special fiber in producing optical components in an easy and fast way. The fabrication involves heating a well-defined portion of fiber with a cyclic moving flame, from 14 to 6.7 mm in amplitude, while applying a tensile force by pulling the fiber with translation stages, each one having sub micrometer resolution. The fiber has an initial *L_c_* = 20 mm, 14 mm of it being tapered down to form the fiber transition regions, and the taper waist. The transition regions can have a controllable shape [30], being linear in the tapered structure presented here.

**Figure 6:**
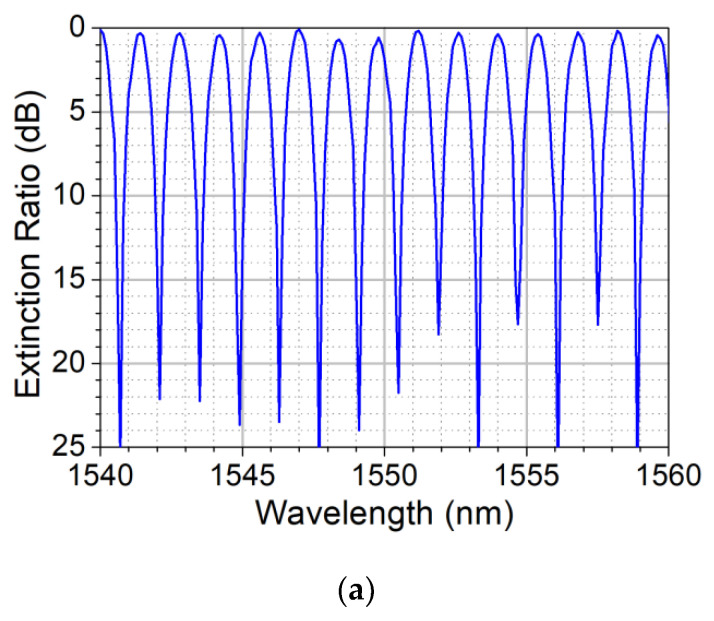

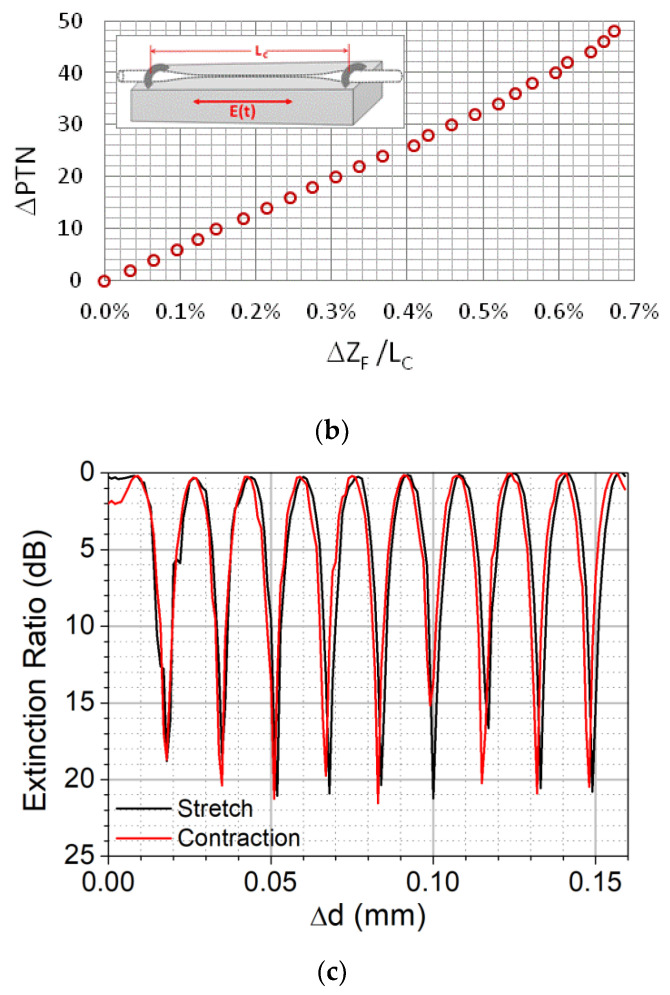
(**a**) Experimental optical transmission spectra of the tapered structure (*L* = 84 mm, *L_c_* = 104 mm) showing FSR = 1.3 nm, insertion loss below 1 dB, and extinction ratio above 17 dB. (**b**) Linear strain sensing response: ΔPTN versus strain Δ*Z_F_*/*L_c_* at 1.55 μm ranging from 0 to 0.7%. (**c**) Symmetric response of the sensor under stretch and contraction for length variation of Δ*d* = 0.15 mm.

Figure 6a shows the experimental optical spectral window, from 1.54 to 1.56 μm, of the taper structure (*L* = 84 mm, *L_c_* = 104 mm) when, after over 950 PTNs, the DCF diameter was reduced from the original 125 μm to 3.9 μm (*ρ_waist_* ~ 2 μm). The FT optical spectra exhibits an insertion loss under 1 dB, an FSR = 1.3 nm, and an extinction ratio above 17 dB. The linear response of this in-line digital strain sensor is presented in Figure 6b, where the ΔPTN is shown as a function of the applied strain (Δ*Z_F_*/*L_c_*). It can be noticed from this figure that the in-line digital strain sensor operates by counting the PTTP = 2ΔPTN when it is under traction/compression along the longitudinal direction, with a digital resolution of 7.4 μm/PTTN. A quasi-symmetric response of this sensor under stretch and contraction up till Δ*d* = 0.15 mm (along its longitudinal direction) presented in Figure 6c allows us to visualize the low backlash. It is important to mention that the transmission and detection of two distinct wavelengths spectrally spaced by an odd multiple of FSR/4 are necessary for sensing and distinguishing compression from traction. For comparison’s sake, the analog response of this same sensor shows a resolution of about 0.7 nm; which is a very high analog resolution for a tapered-based CMZI sensor. Furthermore, this sensor presents a practical digital dynamic range for a myriad of applications.

The proposed digital and linear optical sensing device, with at least two orders of magnitude of dynamic range, operates by counting either PTN or PTTP when submitted to an external force field. The transmission and detection of two wavelengths, spaced apart by an odd multiple of FSR/2, are required to sense traction and compression in this device. 

## 4. Conclusions

We demonstrated an all-fiber digital sensor approach with a linear response based on biconic taper. The tapers-based CMZI were fabricated from a highly DCF using two distinct adiabatic tapering processes, resulting in tapers with a high-quality spectral response. As a proof of concept, we fabricated and characterized an optical axial strain sensor with FSR = 1.3 nm. The sensor is modeled to probe up to 0.7% of strain, returning PTTP = 96, at *λ*_0_ = 1.55 μm, when it is under stress. In such a sensor, when an external force field acts on the optical path of both modes HE_11_ and HE_12_, it produces a linear variation of the PTTP which was used as a discrete/digital response in opposition to the conventional approach (analog), commonly used in interferometric sensors. Also, the axial strain sensor showed a quasi-symmetric response to stretch and compression, thus allowing not only the sensing of these forces but also to distinguish compression and traction. When this sensor was elongated by Δ*L* = 707 µm produced a digital resolution of 7.4 µm/PTTP. Also, the analog response of this same component shows a resolution of about 0.7 nm; which is a very high analog resolution for a taper-based CMZI sensor. 

## Data Availability

Not applicable.
